# Electroresponsive Thiol–Yne Click-Hydrogels for Insulin Smart Delivery: Tackling Sustained Release and Leakage Control

**DOI:** 10.1021/acsapm.4c00911

**Published:** 2024-07-11

**Authors:** Helena Muñoz-Galán, Hamidreza Enshaei, João C. Silva, Teresa Esteves, Frederico Castelo Ferreira, Jordi Casanovas, Joshua C. Worch, Andrew P. Dove, Carlos Alemán, Maria M. Pérez-Madrigal

**Affiliations:** † Departament d’Enginyeria Química, Campus Diagonal Besòs (EEBE), Universitat Politècnica de Catalunya Barcelona Tech, Av. Eduard Maristany 10-14, Barcelona 08019, Spain; ‡ Barcelona Research Center for Multiscale Science and Engineering, EEBE, Universitat Politècnica de Catalunya, C/Eduard Maristany 10-14, Barcelona 08019, Spain; § iBB—Institute for Bioengineering and Biosciences, Department of Bioengineering,Instituto Superior Técnico—Universidade de Lisboa, Avenida Rovisco Pais 1, Lisboa 1049-001, Portugal; ∥ Associate Laboratory i4HB—Institute for Health and Bioeconomy at Instituto Superior Técnico, Universidade de Lisboa, Avenida Rovisco Pais 1, Lisboa 1049-001, Portugal; ⊥ Departament de Química, Física i Ciències Ambientals i del Sòl, Escola Politècnica Superior, Universitat de Lleida, C/Jaume II No. 69, Lleida E-25001, Spain; # School of Chemistry, University of Birmingham, University Rd W, Birmingham B152TT, U.K.; ¶ Institute for Bioengineering of Catalonia (IBEC), The Barcelona Institute of Science and Technology, Baldiri Reixac 10-12, Barcelona 08028, Spain

**Keywords:** electroactive click-hydrogel, thiol–yne nucleophilic addition, PEDOT nanoparticles, insulin delivery, bioelectronics

## Abstract

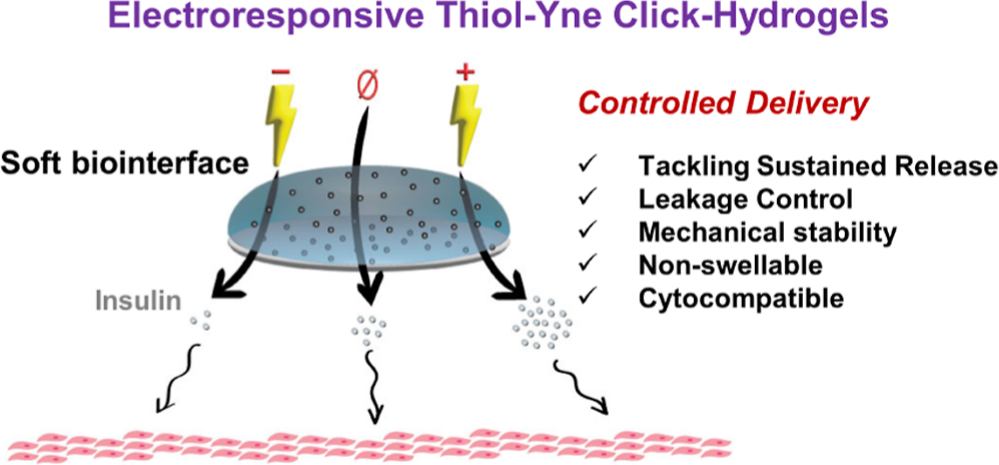

Diabetes is a metabolic disorder caused by the body’s inability to produce or use insulin. Considering the figures projected by the World Health Organization, research on insulin therapy is crucial. Hence, we present a soft biointerface based on a thiol–yne poly(ethylene glycol) (PEG) click-hydrogel as an advanced treatment option to administrate insulin. Most importantly, the device is rendered electroactive by incorporating biocompatible poly(3,4-ethylenedioxythiophene) nanoparticles (PEDOT NPs) as conductive moieties to precisely control the release of insulin over an extended period through electrochemical stimulation (ES). The device has been carefully optimized on account of: (i) the main interactions established between PEDOT- and PEG-based moieties, which have been studied by density functional theory calculations, and reveal the choice of 4-arm PEG precursors as most suitable cross-linkers; and (ii) the concentration of PEDOT NPs in the device, which has been determined considering minimal interference with the gelation process, as well as the resulting morphological, mechanical, electrochemical, and cytocompatible properties of the PEG-based click-hydrogels. Finally, the management over insulin delivery through ES is verified in vitro, with released insulin being detected by high-performance liquid chromatography. Overall, our hydrogel-based device establishes a method for controlled insulin delivery with the potential for translation to other relevant bioelectronic applications.

## 1 Introduction

Diabetes mellitus is a metabolic disorder characterized by hyperglycemia (i.e., elevated levels of glucose in the bloodstream) caused by a dysfunction in the body’s ability to produce or properly use insulin, a hormone secreted by the pancreas that regulates glucose metabolism. In 2021, approximately 537 million adults worldwide suffered from diabetes,^1^ and this number is being projected to increase to 700 million by 2045 according to the World Health Organization (WHO). Besides, the increase in global health expenditure (direct costs) because of diabetes has grown from US $232 billion in 2007 to US $966 billion in 2021 for adults aged 20–79 years.^2^

A proper management of this disease is essential to prevent serious health issues. The usual treatment typically involves a combination of lifestyle modifications, such as regular exercise and a healthy and balanced diet. In most severe cases, insulin administration is prescribed through injections or insulin pumps.^3−5^ Therefore, research on insulin therapy is vital not only to improve the quality of life of diabetic patients, but also on a larger scale to positively impact the global economy by reducing the healthcare direct and indirect costs associated with the treatment of diabetes.

At the moment, the market of digital devices for insulin release is currently expanding on account of the increasing number of patients using such technology to treat their medical condition, as well as decreasing production costs. According to a recent study published by Transparency Market Research (TMR), this commercial technology is thriving and is anticipated to generate a revenue valuation of US $2082.3 million by 2025.^6^ On that account, the development of advanced and upgraded devices is a matter of both social and economic interest to all.

On the one hand, state-of-the-art insulin delivery research is focused on optimizing closed-loop systems,^7^ with high dosage precision on insulin delivery and accurate reproducibility on glucose detection, while, on the other hand, ensuring proper device biocompatibility, insulin stability, and implementation of minimal invasive approaches.^8^ To do so, numerous approaches have been recently reported, which include insulin encapsulation into nanoparticles^9,10^ and the use of hydrogels as embedding systems.^11,12^ Alternatively, electrostatic complexes have also been used to deliver insulin as opposed to systems based on physical entrapment.^13^ With an isoelectric point of 5.4, insulin is slightly negatively charged at pH 7.4. Hence, it can be stabilized by polycations at physiological pH, whereas, at lower pH values, such electrostatic interactions are disrupted, which allow insulin to be released. Although such principles can be explored to design a potential delivery approach, the lasting effect and uncontrolled leakage of insulin still need to be optimized.

To achieve the desired concentration of insulin release, which is a challenging task, several polymeric matrices have been proven to be biocompatible and biodegradable, such as chitosan^14^ or alginate.^15,16^ Most importantly, however, insulin release requires a smarter and more controlled delivery, which is expected to take into account the patient’s glucose level in blood. Stimuli-responsive systems represent an efficient strategy to tackle this issue and promote a better glycemic control. In hydrogel-based devices, the variety of stimuli that trigger insulin release include physiological temperature,^17,18^ pH,^19−21^ electrical current,^22−26^ dynamic covalent bonding,^27^ or glucose itself,^16,28−30^ among others.^31^

More than two decades ago, in 2001, Sharpless and coworkers devised click chemistry as a set of powerful, highly reliable, and selective reactions to produce functional molecules in a straightforward and accessible manner.^32^ Since then, the development of methods in the area of click chemistry has been continuously developing,^33^ and, most noticeably, being applied across scientific disciplines (e.g., pharmaceutical and biotechnological industry, material science, polymer chemistry, bioconjugation, and biolabeling, among others).^34−38^ For instance, in the field of bioelectronics, hydrogels are conceived as soft biointerfaces able to bridge the gap between biology and electronics to produce devices with advanced features.^39^ Conductive hydrogels act as important functional elements in specific biodevices, such as sensors, actuators, smart adhesives, electrically stimulated platforms for drug delivery and tissue regeneration.^40^ Moreover, if derived from click chemistry approaches, their “click” nature yields additional advantages.

In that regard, among the metal-free click tools that are currently available,^41^ the nucleophilic thiol–yne addition reaction^42,43^ has become a relevant option to produce cytocompatible hydrogels based on poly(ethylene glycol) (PEG) for biotechnological applications, such as platforms for cell mechanotransduction studies,^44^ with outstanding mechanical performance,^45,46^ nonswellable properties,^47^ as well as self-healing and stretchable response,^48^ alone or in combination with biorelevant polysaccharides (e.g., hyaluronic acid).^49^ Most importantly, PEG derivatives not only have been reported as biocompatible and suitable for tissue engineering but also as effective carriers for drug release,^50,51^ including insulin.^52^

Considering all that is mentioned above, herein we take advantage of the clickable nature of thiol–yne PEG-based click-hydrogels and turn them into a soft biointerface for an optimized insulin delivery. Most importantly, we rendered them electroactive by incorporating biocompatible poly(3,4-ethylenedioxythiophene) nanoparticles (PEDOT NPs) as conductive moieties to precisely control the release of insulin over an extended period of time through electrical stimulation. To the best of our knowledge, this is the first time that electroresponsive nucleophilic thiol–yne click-hydrogels are reported. We hypothesized that the nonswellability and mechanical robustness of these click-hydrogels (i.e. high compressive strength between 0.1 and 0.4 MPa with little swelling over time), in addition to their biocompatibility and straightforward fabrication, would result in adequate functional elements in digital devices for insulin release. Moreover, their hydrophobic character ensures no bulk degradation for at least 15 days after immersion in an aqueous environment under physiological conditions. Therefore, in pursuit of high-impact materials for improving human health, electroresponsive thiol–yne PEG-based click-hydrogels are presented as unique conductive polymeric matrices designed to control insulin delivery (i.e., hyperglycemic management) and, in turn, suitable for other relevant bioelectronic applications beyond the one reported herein.

## 2 Results and Discussion

**Figure 1:**
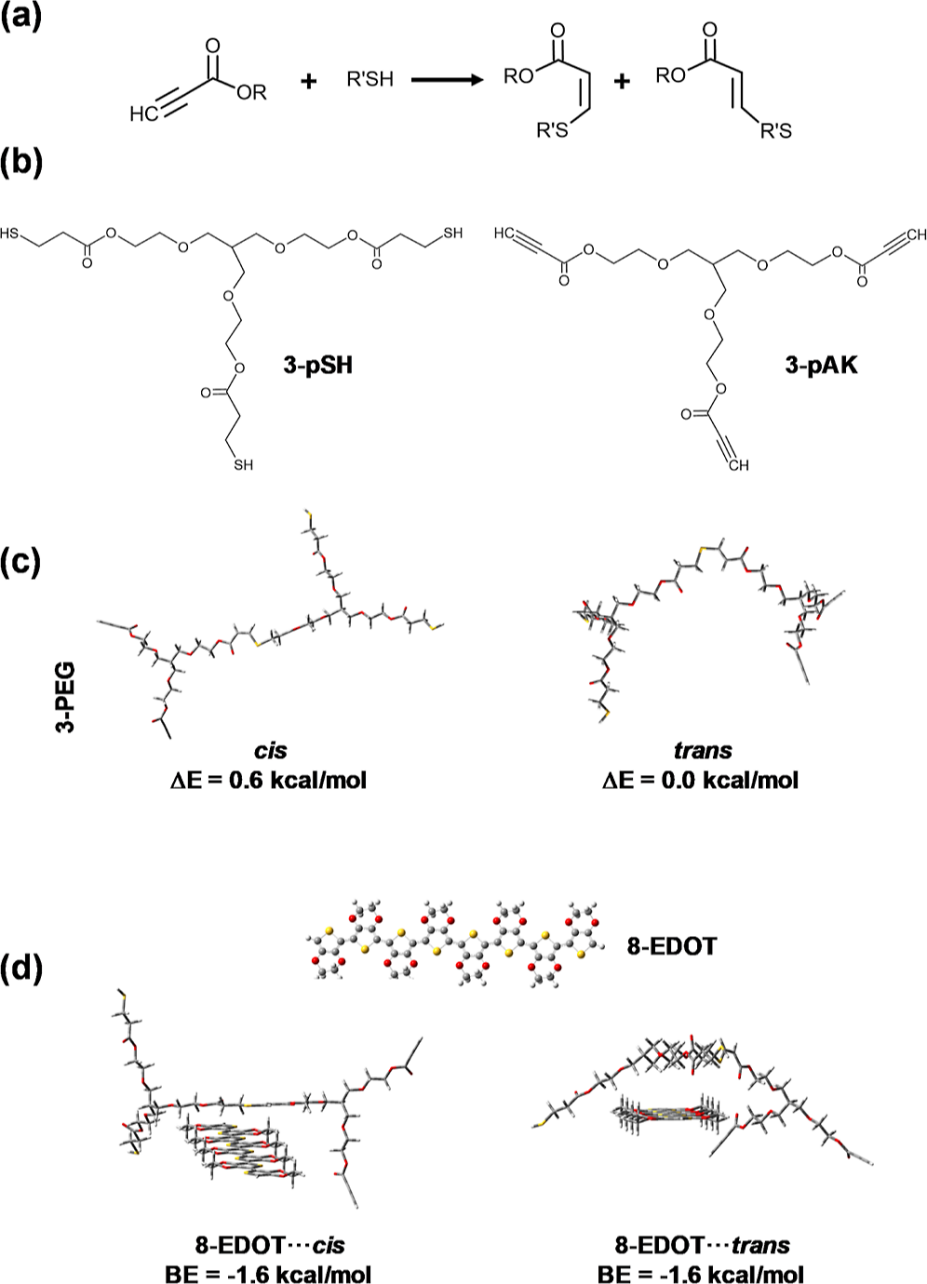
Theoretical study of interactions between PEDOT and representative 3-arm PEG-based moieties. (a) Scheme of the nucleophilic thiol–yne addition reaction. (b) Chemical structures of the 3-arm thiol and alkyne functionalized PEG precursors (3-pSH and 3-pAK, respectively). (c) Cross-linked 3-arm PEG unit considering both cis and trans arrangements. (d) The most stable structure for each 8-EDOT···cis and 8-EDOT···trans complex.

First, to understand the main interactions established between PEDOT- and PEG-based moieties, as well as the compounds formed by the nucleophilic thiol–yne addition reaction (Figure 1a), density functional theory (DFT) calculations were conducted considering representative molecules. Specifically, models for 3-arm and 4-arm alkyne- and thiol-functionalized PEG molecules, which contained one repeating unit and a molecular weight in the range between 394 and 679 g/mol, and EDOT oligomers, which contained several repeating units, were used. Then, as per the theoretical study results, 4-arm alkyne- and thiol-functionalized PEG precursors (2 kg/mol) were synthesized by Fischer esterification and subsequently mixed in a 1:1 molar ratio of alkyne to thiol polymer precursors at 21 °C using phosphate buffered saline (PBS) solution as solvent and a solids content of 15 wt %, while PEDOT NPs were prepared by oxidative emulsion polymerization and introduced prior gelation (6.2 mg/mL). A mixture of insulin and PEDOT NPs was solvent-casted on top of the working electrode (WE), and then covered with the click-hydrogel solution; gelation took place in situ within less than a minute. Then, the effect of PEDOT NPs on the morphological, mechanical, electrochemical, and cytocompatibility properties of the PEG-based click-hydrogels was determined, while their suitability as insulin delivery systems was verified. Finally, the management over insulin delivery by electrochemical stimulation (ES) was optimized, with released insulin being detected by high-performance liquid chromatography (HPLC).

### 2.1 Theoretical Study of Interactions Between PEDOT and PEG-Based Moieties

The molecular geometries of the representative 3-arm thiol and alkyne functionalized PEG precursors (3-pSH and 3-pAK, respectively, Figure 1b) were optimized in the vacuum using DFT calculations at the B3LYP/6-311++G(d,p) level and, subsequently, reoptimized in aqueous solution using the polarizable continuum model (PCM), which is a well-established self-consistent reaction field method. Then, the two systems were cross-linked considering both cis and trans arrangements to form the 3-arm PEG unit (Figure 1c). Geometry optimizations in aqueous solution indicated that the trans arrangement was slightly more favored than the cis (Figure 1c). Then, simple model systems involving a previously optimized PEDOT chain with eight repeat units and a charge of +0.5 per repeat unit complexed to the 3-arm PEG unit arranged in cis and trans configuration (8-EDOT···cis and 8-EDOT···trans, respectively). The half positive charge used for each PEDOT repeat unit, which reflects the characteristic delocalized electronic distribution of heterocyclic conducting polymers, was taken from previous experimental measures of the doping level.^53^ In order to search for the most favorable interaction between the model molecules involved in 8-EDOT···cis and 8-EDOT···trans complexes, different relative orientations were considered as starting points for geometry optimizations (i.e., at least five different starting points were considered for each complex). The most stable structure for each complex is shown in Figure 1d. It is worth noting that the two structures were practically isoenergetic. Also, the binding energy (BE), which measures the interaction energy between the two model molecules, was identical (BE = −1.6 kcal/mol).

**Figure 2:**
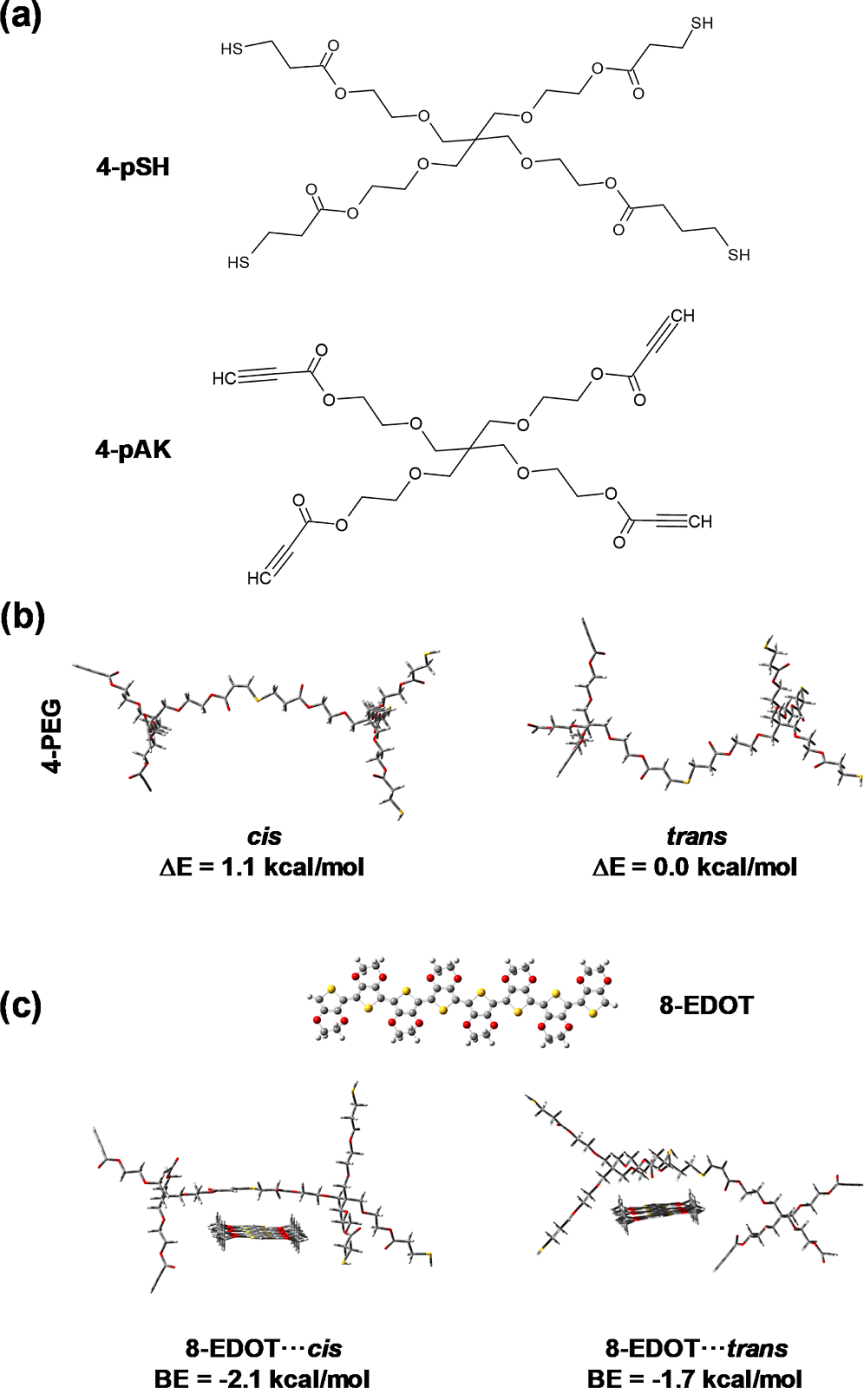
Theoretical study of interactions between PEDOT and representative 4-arm PEG-based moieties. (a) Chemical structures of the 4-arm thiol and alkyne functionalized PEG precursors (4-pSH and 4-pAK, respectively). (b) Cross-linked 4-arm PEG unit considering both cis and trans arrangements. (c) The most stable structure for each 8-EDOT···cis and 8-EDOT···trans complex.

The same procedure was applied for evaluating the stability and binding affinity of the 4-arm model structures (Figure 2). In such case, the trans arrangement of 4-arm PEG unit derived from the reaction of the 4-arm thiol and alkyne functionalized precursors (4-pSH and 4-pAK, respectively, in Figure 2a), was more stable than the cis by 1.1 kcal/mol (Figure 2b). However, this energy difference is not representative of the hydrogel system since the favorable interactions and/or steric effects associated with the molecular chain propagation effects are not considered in the modeled structure, which simply consists of a 4-arm PEG unit. On the other hand, inspection of the results obtained for the most stable 8-EDOT···cis and 8-EDOT···trans complexes (Figure 2c) indicates that the incorporation of the PEDOT chain reduces this energy by half. This has been attributed to the affinity between the two interacting molecules, which results in an enhancement of the BE not only with respect to the 8-EDOT···trans complex, but also with respect to the two complexes involving the 3-arm PEG unit (Figure 1d). Overall, results displayed in Figures 1 and 2 reflect a favorable interaction between PEDOT and the two examined PEG-based click-hydrogels. However, the interaction is more stable for the 4-arm hydrogel complex than for the 3-arm hydrogel one, which suggests that the former could provide better electroresponsive properties.

### 2.2 Preparation and Characterization of Electroresponsive Thiol–Yne PEG Click-Hydrogels

**Figure 3:**
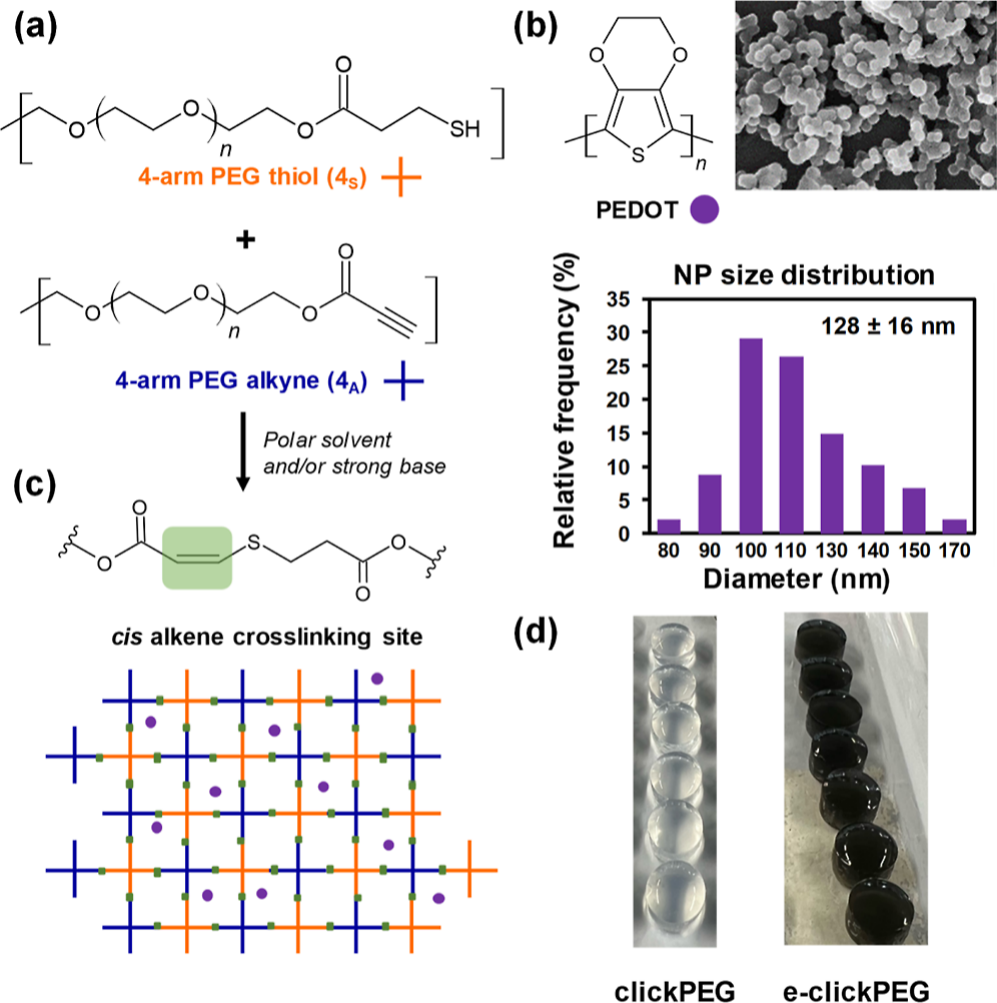
Preparation of e-clickPEG hydrogels. (a) 4-Arm alkyne- and thiol-functionalized PEG precursors (4_A_ and 4_S_, respectively) were synthesized by Fischer esterification. (b) PEDOT NPs as conductive element in the system (SEM morphology and size distribution). (c) Scheme of the resulting hydrogel cross-linked by cis alkene bonds. (d) Photographic images of the resulting conductive e-clickPEG hydrogels (black color) in contrast to the blank system (without PEDOT NPs; clickPEG hydrogel).

Clickable thiol–yne PEG-based hydrogels have been applied in this study as the core in insulin delivery devices where release is controlled by ES. Based on the theoretical study, to render the hydrogel system electroactive, biocompatible PEDOT NPs were chosen and mixed prior to gelation. 4-Arm alkyne- and thiol-functionalized PEG precursors (4_A_ and 4_S_, respectively, Figure 3a) were synthesized by Fischer esterification as previously reported.^47^ Then, 4_A_ (75.0 mg) and 4_S_ (79.9 mg) were dissolved separately in PBS (0.5 mL) at 21 °C (1:1 molar ratio of alkyne-to-thiol polymer). The conductive component, which is PEDOT NPs (Figure 3b), was prepared by oxidative emulsion polymerization^54^ with a narrow size distribution, corresponding to an average diameter of 128 ± 16 nm (*n* = 100), in good agreement with previous results,^23^ and suspended in each PBS PEG-precursor solution at a concentration of 6.2 mg/mL. After stabilizing the PEDOT NPs by vortexing for a few seconds, both solutions were mixed, and gelation was allowed to proceed at ambient conditions. PEG total solid content was optimized at 15 wt % to ensure (i) gelation under 1 min (40 s by vial tilting method) and (ii) formulation of hydrogels with high PEDOT NPs content (Figure 3c). Hereafter, thiol–yne PEG-based click-hydrogels containing PEDOT NPs will be referred to as e-clickPEG hydrogels, while those without conductive moieties will be referred to as clickPEG hydrogels.

Following this procedure, stable hydrogels were obtained with excellent reproducibility, and those containing PEDOT NPs exhibited a blackish color (Figure 3d). The gel fraction (GF, Table 1) for the blank system with no PEDOT NPs was determined to be 82 ± 0.3%, which is in good agreement with previous reported values,^47^ and evidence the high efficiency of the cross-linking reaction. Upon addition of NPs, the GF decreased to 67 ± 0.4%, which is ascribed to a slightly less efficient cross-linking because of the presence of the NPs during gelation, as well as their loss during the washing steps of the procedure, which affected the GF calculation. Despite this difference, e-clickPEG hydrogels were mechanically stable. The equilibrium water content (EWC) determined after 24 h of immersion revealed that the presence of PEDOT NPs did not significantly affect the ability of the PEG-based thiol–yne click-hydrogels to hold water. For clickPEG and e-clickPEG hydrogels, EWC values were determined to be 71 ± 0.6 and 73 ± 2.3%, respectively.

**Table 1:** Gelation Fraction (GF), Swelling Kinetics EWC and Mechanical Properties (Measured in Compression) Determined for clickPEG and e-clickPEG Hydrogels^a^

	clickPEG	e-clickPEG
GF (%)	82 ± 0.3	67 ± 0.4
EWC (%)	71 ± 0.6	73 ± 2.3
*E* (Young’s modulus, kPa)	49 ± 16	37 ± 8
Strength at break (kPa)	104 ± 18	110 ± 13
Strain at break (%)	49 ± 6	54 ± 8

a Errors = s.d. with *n* = 6–8.

**Figure 4:**
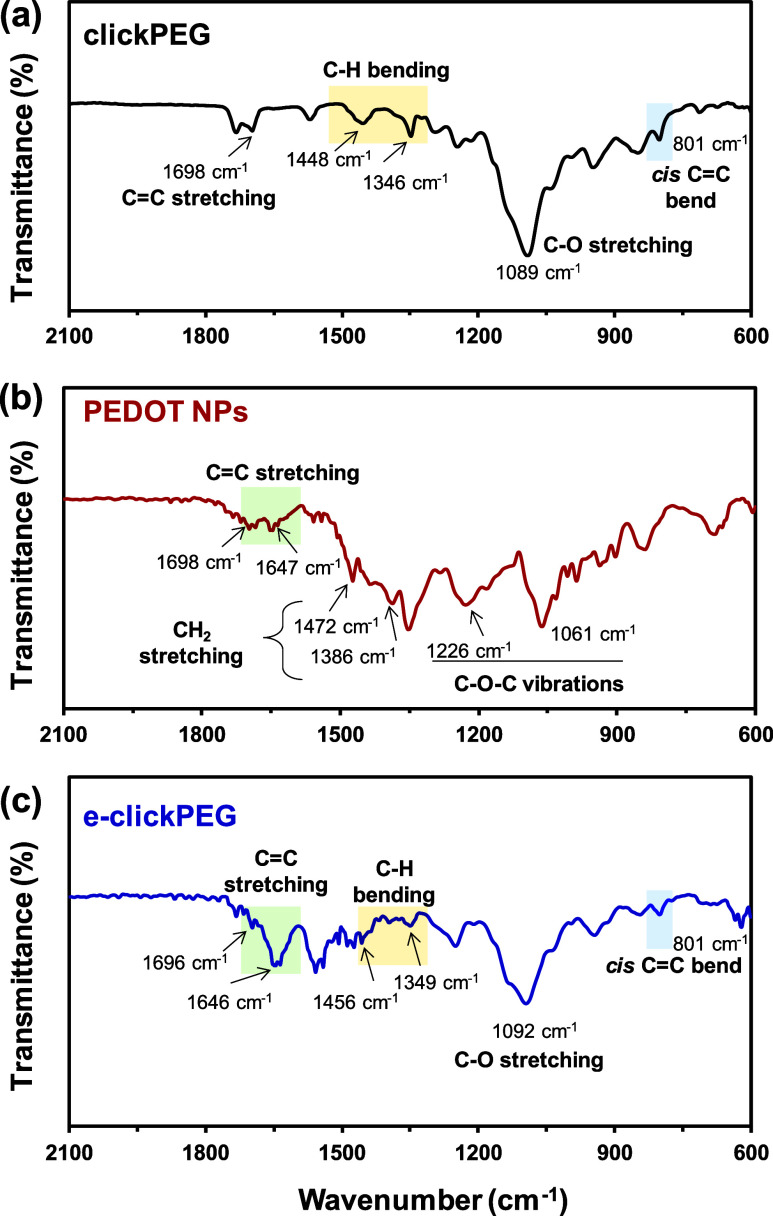
FT-IR spectra obtained for (a) thiol–yne clickPEG hydrogels; (b) PEDOT NPs, and (c) e-clickPEG hydrogels.

The chemical composition of e-clickPEG hydrogels, as well as each component separately (PEDOT NPs and clickPEG hydrogels), was determined by FT-IR spectroscopy (Figures 4 and S1). In the spectrum of clickPEG hydrogels (Figure 4a), peaks at approximately 1100, 1350–1450, 1690, 1730 and 2870 cm^–1^ corresponded to the C–O stretching of the ether group, –CH bending, C=C stretching, C=O ester stretching, and –CH stretching, respectively, in good agreement with previous work.^44,55−57^ Regarding PEDOT NPs, the most characteristic peaks were related to the ether bond and thiophene ring: C=C stretching at 1698 and 1647 cm^–1^, CH_2_ stretching at 1472 and 1386 cm^–1^, and C–O–C vibrations at 1226 and 1061 cm^–1^ (Figure 4b).^58^ Upon loading PEDOT NPs, the signal of C=C stretching from PEDOT appeared in e-clickPEG hydrogels overlapping with the C=C stretching band from the clickPEG hydrogel. The other bands remained unaltered with no significant shift being appreciated, which shows that specific interactions with charged (oxidized) PEDOT chains were not established and, furthermore, verifies the incorporation of PEDOT NPs. Most importantly, regarding the stereochemistry of the gels, the signal attributed to the PEG cis C=C bend appeared at ca. 802 cm^–1^ both in clickPEG and e-clickPEG hydrogels, which indicates that the cis content was high, as expected (i.e., polar solvents yield 100% cis content),^42,44^ regardless of the presence of PEDOT NPs (Figure 4c).

**Figure 5:**
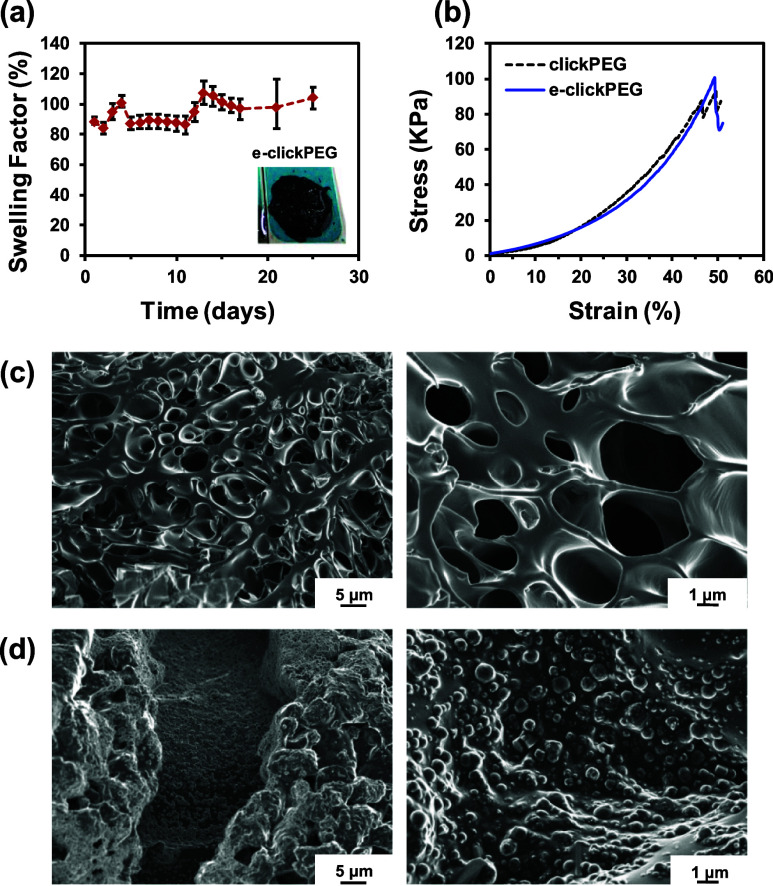
(a) Swelling factor profile of e-clickPEG hydrogels immersed in PBS and incubated at 37 °C in an orbital shaker incubator at 80 rpm. Image shows an e-clickPEG hydrogel after 25 days of immersion. (b) Representative stress–strain compressive curves recorded for both clickPEG and e-clickPEG hydrogels. SEM micrographs obtained for (c) clickPEG hydrogels and (d) e-clickPEG hydrogels. Scale bar of 5 and 1 μm in left and right images, respectively.

To exploit e-clickPEG hydrogels as soft biointerfaces for precisely controlling insulin delivery, both the nonswelling profiles, as well as the mechanical integrity and robustness of clickPEG hydrogel need to be preserved after PEDOT NPs loading. After immersion in an aqueous environment under physiological conditions, the increase in volume was controlled and, accordingly, swelling was suppressed. e-clickPEG hydrogels shrunk moderately down to 84% after 24 h and then recovered and remained at around 100% for at least 25 days with no signs of bulk degradation (Figure 5a). Such stability and volume retaining allow for an adequate application of the device, whose form will not be altered by swelling, thus facilitating its design and ensuring long-term usage, with special effect on its mechanical performance.^47^

The mechanical properties of the e-clickPEG hydrogels were assessed through uniaxial compression (Figure 5b) using cylindrical hydrogels (5 mm in height and 8 mm in diameter). After adding PEDOT NPs, both the compressive strength and the strain at break increased slightly in comparison to the blank system (clickPEG hydrogels) although with no significant differences (Table 1). Overall, the presence of the conductive NPs in e-clickPEG hydrogels did not affect the robustness and excellent mechanical performance of these click hydrogels.^44,47^ Specifically, e-clickPEG hydrogels failed at 54 ± 8%, while hydrogels without PEDOT NPs displayed a strain at break of 49 ± 6%. Regarding compressive strength, the values recorded for clickPEG and e-clickPEG hydrogels were 104 ± 18 and 110 ± 13 kPa, respectively. In contrast, the compressive Young’s modulus decreased from 49 ± 16 kPa down to 37 ± 8 kPa after adding the PEDOT NPs. Hence, conductive hydrogels are softer, probably because of the electroactive NPs interfering with the cross-linking of the PEG network (also observed previously with the calculated GF values) and, thus, producing less stiff materials.

In terms of morphology, for clickPEG hydrogels, the porous structure observed is smooth and displays pores with a range of sizes between 0.5 and 2 μm, which corresponds to the growth of ice crystals during the freezing process (Figure 5c). The incorporation of PEDOT NPs did alter significantly the porous structure observed for clickPEG hydrogels in that the surface and walls of the pores are covered with NPs, which are densely and homogeneously distributed (Figure 5d). The size of the conductive particles, which are clearly distinguishable, vary from 0.2 to 1.7 μm, being the average 0.7 ± 0.3 μm, sizes that are 5–6 higher than the one of PEDOT NP characterized after their synthesis. Hence, during either the gel formation or the freeze-drying step, NPs agglomerate to yield larger structures. Despite this, they are able to form effective electrochemical and conductive paths (see below) as particles are well connected to each other without compromising any relevant feature that is needed for smart insulin delivery, that is, mechanical stability and adequate swelling.

**Figure 6:**
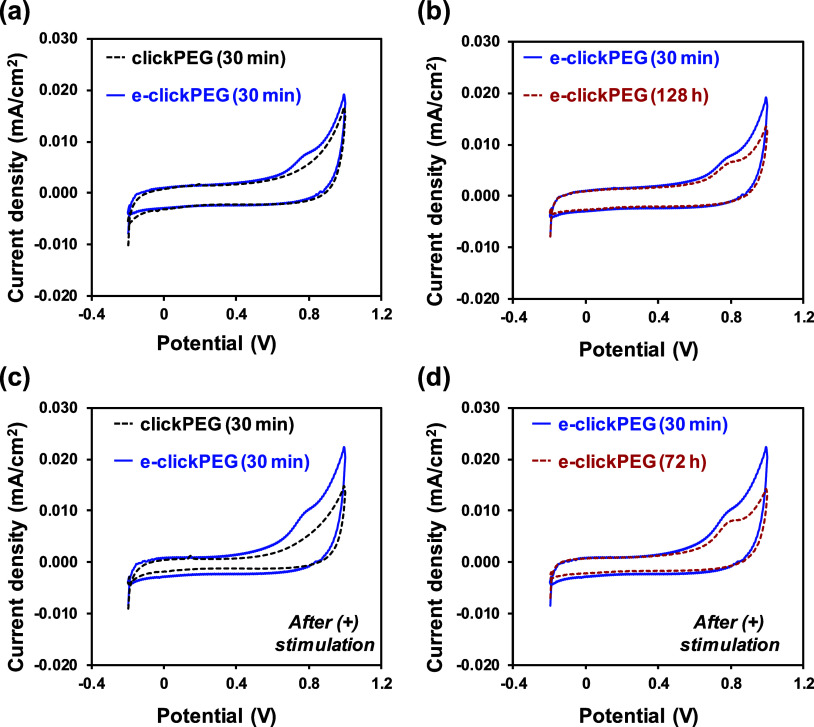
Electrochemical response of the prepared systems. Representative third CV scan recorded for blank thiol–yne clickPEG and conductive e-clickPEG hydrogels after being immersed in PBS solution for (a) 30 min and (b) 128 h (∼5 days). Representative third CV scan recorded for blank thiol–yne clickPEG and conductive e-clickPEG hydrogels after being electrically stimulated (+0.6 V for 100 s) at time options (c) 30 min and (d) 72 h (3 days).

Finally, the electrochemical response of clickPEG and e-clickPEG hydrogels was determined by cyclic voltammetry (CV, Figure 6). For each system (*n* = 3), three CV cycles were run in PBS (pH = 7.4) at room temperature from −0.2 to 1.0 V (initial/final and reversal potentials, respectively) at 50 mV/s to determine the effect of adding PEDOT NPs on the electrochemical response of the hydrogels, as well as the influence of immersion time.

Comparison between the third cyclic voltammogram recorded for clickPEG and e-clickPEG at an immersion time of 30 min reveals that the loading of PEDOT NPs increases the electrochemical activity, as it is observed by the increment in the voltammetric area of e-clickPEG with respect to the blank hydrogel (Figure 6a). Additionally, an oxidation peak at around ∼0.75 V is clearly detected and, whereas the corresponding reduction peak (∼0.8 V) is less noticeable, we ascribed the presence of this redox pair in the recorded potential window to the formation of polarons in the PEDOT chains.^59,60^ Most importantly, the electroactive response displayed by e-clickPEG hydrogels is retained after an immersion time of 5 days (Figure 6b). Although the voltammetric area diminished slightly with immersion time, the redox behavior of PEDOT remains unalterable, which ensures their electroactivity for long periods and, consequently, their interaction with insulin during electrostimulated-controlled release.

### 2.3 Design of NPs + Insulin/e-clickPEG Devices: Electrochemical Management of Insulin Delivery

Insulin delivery research faces an important challenge, which is the uncontrolled leakage of insulin from hydrogel-based devices. Moreover, being able to control the concentration of released insulin allows for an improved glycemic control. Our stimuli-responsive device aims to tackle both aspects by the use of e-clickPEG hydrogels and ES.

**Figure 7:**
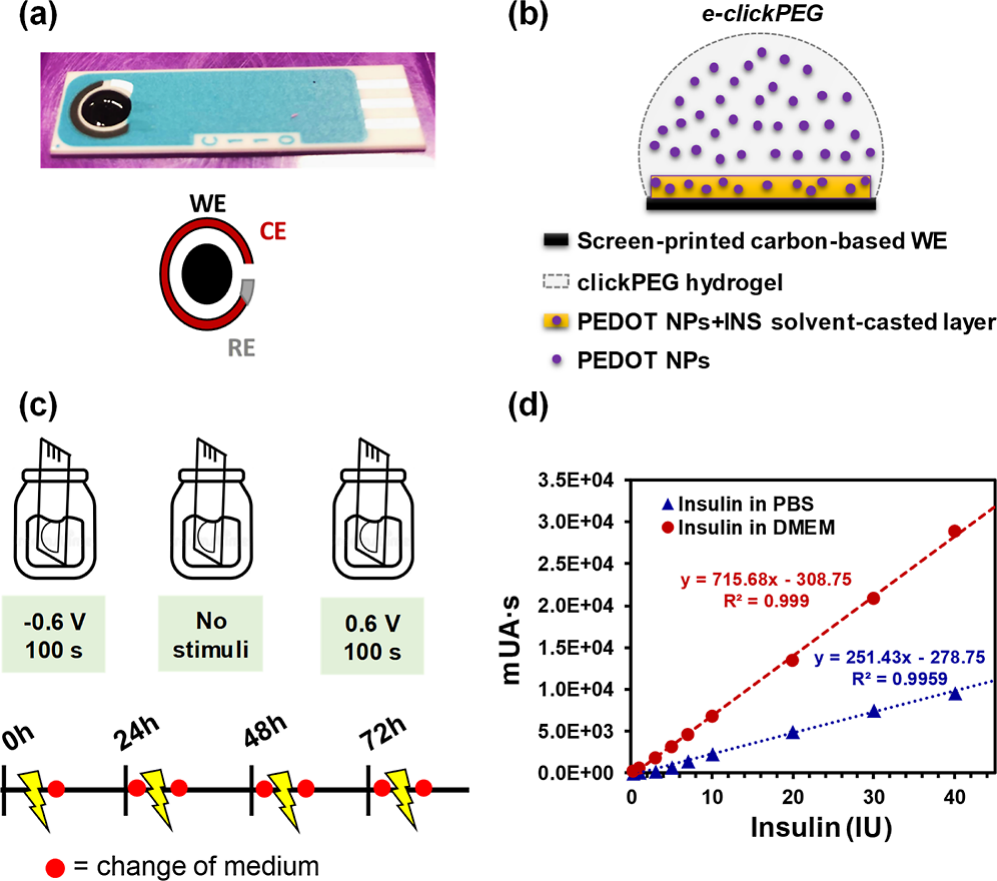
Electrochemical management of insulin delivery from NPs + insulin/e-clickPEG devices. (a) Image of the screen-printed electrode used for ES testing. (b) Schematic of the NPs + insulin/e-clickPEG device prepared on top of the WE. (c) Conditions considered for the electrochemical stimulated insulin release. Every 24 h, 2 mL of media were collected and replaced before and after the stimulation event. (d) Calibration curves obtained by HPLC for insulin detection both in PBS and cell culture medium.

Specifically, the insulin release system has been designed as follows: first, a mixture of PEDOT NPs and insulin was stabilized in 0.1 M PBS and then drop-casted onto a screen-printed electrode (WE) to yield a thin coating layer (NPs + insulin). Once dried, the e-clickPEG hydrogel was formed on top of it and let to gel for 15 min before any release study (Figure 7a,b). The final device will be referred to as NPs + insulin/e-clickPEG. In this configuration the e-clickPEG hydrogel acts as an embedding 3D porous polymeric matrix that protects insulin from enzymatic degradation, thus ensuring a longer stability, as well as slowing down its rapid diffusion rate if uncovered.

To explore the electrochemical management of insulin delivery from NPs + insulin/e-clickPEG devices, they were immersed in 2 mL of release media (i.e., PBS or Dulbecco’s modified Eagle medium, DMEM) and exposed to a constant stimulation potential for 100 s per day during 4 days (*n* = 6). Three different trigger conditions were considered: a positive voltage (0.6 V), a negative voltage (−0.6 V), and no voltage. Every 24 h, the 2 mL of media were collected and replaced before and after the stimulation event, respectively (Figure 7c). Insulin concentration was determined by HPLC after performing the corresponding calibration curves (Figure 7d). To verify the adequacy of NPs + insulin/e-clickPEG devices to be used in a biological context (as potential subcutaneous skin patches, for instance), electrochemical controlled release experiments were also conducted in the presence of fibroblasts (L-929 cell line; mouse fibroblasts; subcutaneous connective tissue; adipose).

The objective of electrochemically stimulating PEDOT NPs within the click-hydrogel and the NPs + insulin layer to control insulin delivery is 2-fold. On the one hand, we hypothesize that external ES affects the oxidation degree of PEDOT chains, which alters PEDOT···insulin and counteranion···insulin electrostatic interaction. Accordingly, changes in those interactions contribute to either retain insulin within the NPs + insulin layer or, on the contrary, facilitate its release toward the solution media through the PEG matrix. On the other hand, similarly, PEDOT NPs found inside the e-clickPEG hydrogel also expand and contract on account of the creation/disappearance of positive charges and the movement in and out of counter-anions during ES. Hence, depending on the type of stimulus, PEDOT NPs in the PEG matrix act as anchoring insulin points, which slow down the diffusion of insulin already released from the coating layer.

**Figure 8:**
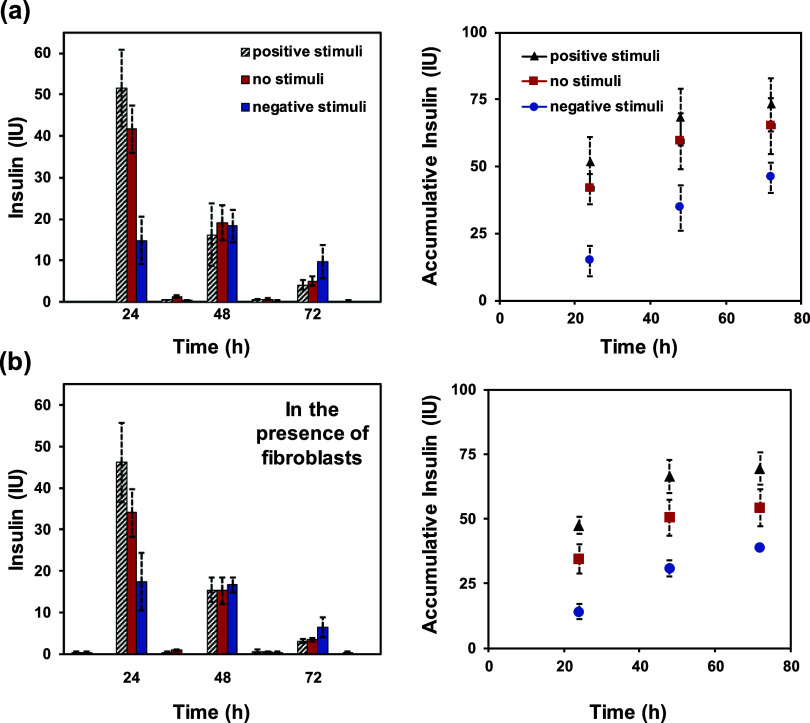
Absolute (left) and accumulative (right) values of insulin units released from NPs + insulin/e-clickPEG devices (*n* = 6) at specific time intervals depending on the electrochemical stimuli applied: (a) in DMEM media and (b) in DMEM media in the presence of fibroblasts cells.

The insulin delivery profile from NPs + insulin/e-clickPEG devices stimulated in DMEM with and without the presence of cells revealed the effectiveness of the constant positive stimulation (+0.6 V) to trigger the release of insulin, while a negative stimulation (−0.6 V) inhibited insulin from being leaked through the system. The insulin delivery profile from NPs + insulin/e-clickPEG devices not being submitted to stimulation was considered as a reference value that represents the passive insulin release only promoted by diffusion and affected by any change in the PEG polymer matrix. In this case, insulin release reached its maximum value at the end of the test period (i.e., 4 days). Hence, in DMEM media, the application of no stimulus resulted in an accumulative insulin release of 65 ± 10% of the total insulin loaded on the hydrogel, whereas positive or negative stimuli did increase (73 ± 9.8%) or decrease (46 ± 5.7%) insulin delivery, respectively (Figure 8a). A similar trend, with values slightly lower, was obtained for NPs + insulin/e-clickPEG devices stimulated in the presence of fibroblasts in DMEM culture media (Figure 8b). Accumulative insulin release values were determined to be 69 ± 6.4, 54 ± 7.1, and 38 ± 1% for positive, none, and negative stimulation, respectively. Insulin degradation produced by cell-driven enzymatic proteases might explain the difference in values. Besides, it was verified that the application of a constant positive stimulation (+0.6 V) for 100 s (at stimulation time points of 30 min and 72 h, Figure 6c,d) did not alter the redox behavior of PEDOT NPs, which completely retained their electroactivity.

Which mechanism is behind such performance? Insulin has a negative net charge at physiological pH values. Hence, when the system is submitted to a constant positive potential, the oxidation process taking place further creates positive charges in the PEDOT chains that force the entrance of counter-anions to compensate for the overall charge. These new anions display a shielding effect that weakens any PEDOT···insulin electrostatic interactions previously established. Also, as oxidation progresses leading to additional anions to enter into the PEDOT layer, repulsive sites between counter-anions and insulin are being created, thus forcing insulin to being released. In contrast, when the system is submitted to a constant negative potential, the doping level of the conjugated polymer film decreases and, therefore, the charge of PEDOT chains at the interface becomes smaller and the corresponding counter-anions that act as dopants tend to be expelled from the matrix. As this happens, insulin is retained in the NPs + insulin layer balancing the change in electrostatic interactions due to counter-anions moving out to the solution media. As the negative stimulation lasts only for 100 s, this amount of time is sufficient to prevent insulin from leaving the layer before any passive diffusion process starts.

Overall, if further optimized as a closed-loop system electrochemically activated, NPs + insulin/e-clickPEG devices allow for insulin sustained release and, most importantly, leakage control. Not only that, but an additional PEDOT layer can be designed to act as a glucose sensor as well, thus rendering the device smarter in that its insulin delivery response is linked to the patient’s sugar level.

### 2.4 Cytocompatibility of NPs + Insulin/e-clickPEG Devices for Insulin Therapy

**Figure 9:**
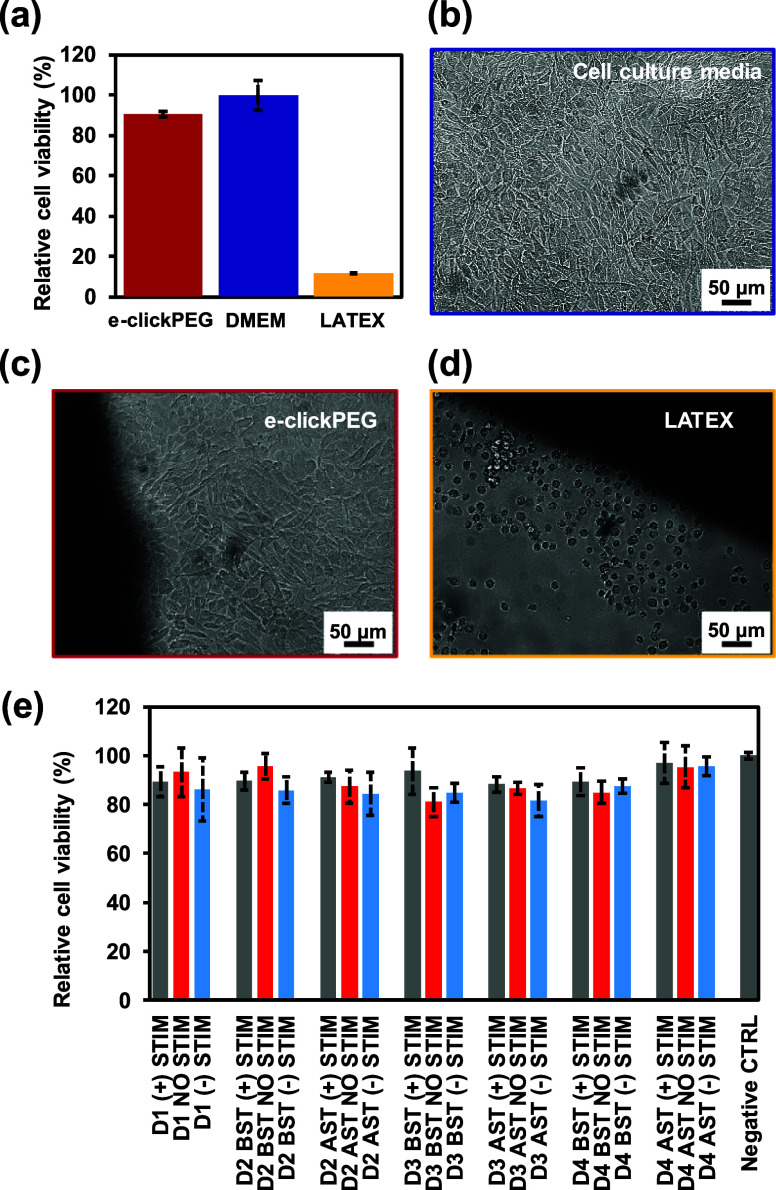
Cytocompatibility of NPs + insulin/e-clickPEG devices. (a) Relative cell viability of L-929 mouse fibroblasts cultured in extracts from e-clickPEG hydrogels, DMEM culture medium and latex. (b–d) Optical images of L-929 cells cultured in direct contact with the tested systems: (b) control, (c) e-clickPEG hydrogels, and (d) latex. Scale bar of 50 μm in all images. (e) Relative cell viability of L-929 mouse fibroblasts cultured in electro-stimulated media, either positive (+), negative (−), or no stimuli (NO) at different time points [day (D) 1, 2, 3, and 4] before (BST) and after stimulation (AST).

The cytocompatibility of e-clickPEG hydrogels was assessed using L-929 mouse fibroblasts, following the ISO 10993-5 and ISO 10993-12 guidelines, by both an indirect extract test (Figure 9a) and direct contact test (Figure 9b–d). For both tests, L-929 cells cultured in cell media (DMEM + 10% FBS + 1% anti–anti) under standard conditions were used as negative control, while those cultured in the presence of latex were used as a positive control for cell death. Extracts from e-clickPEG hydrogels were shown to be cytocompatible, presenting high relative cell viability (90.5 ± 1.5%) in the [3-(4,5-dimethylthiazol-2-yl)-2,5-diphenyl tetrazolium bromide] (MTT) assay results (Figure 9a), which is in accordance with previous studies using PEG thiol–yne click hydrogels.^44^ Besides, in good agreement with the quantitative data, optical images of cells cultured for 72 h in direct contact with the conductive hydrogel revealed no cytotoxicity and favorable biocompatibility (Figure 9c). Cells displayed a healthy density and a typical spindle-like morphology, and there was no evidence of cell death or any inhibition halo effect. Hence, both the materials used, as well as the preparation process, ensure viable cells, with safety and biocompatibility not being affected.

**Figure 10:**
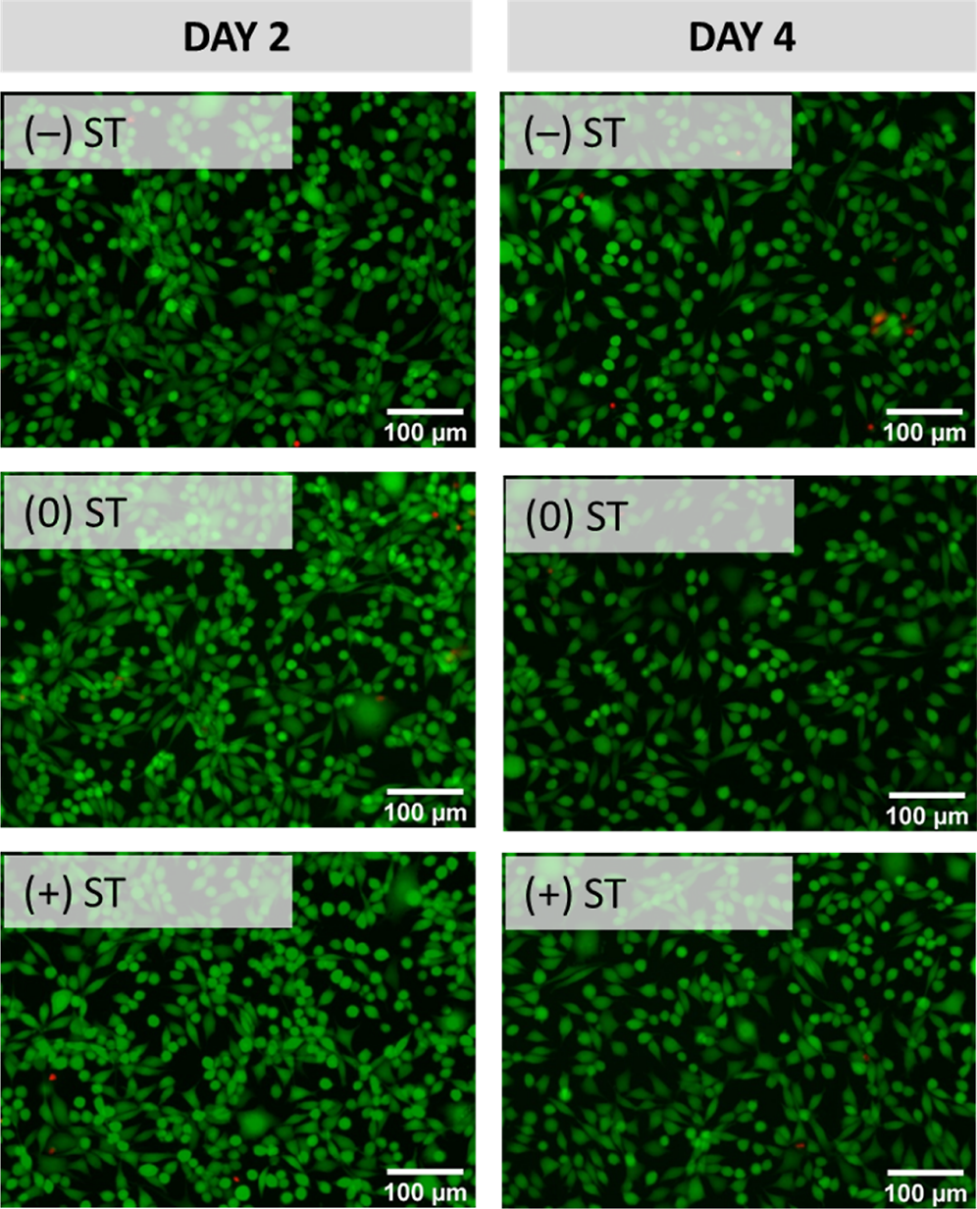
Cytotcompatibility of leachable products from insulin delivery devices. Fluorescence microscopy images of L-929 mouse fibroblasts cultured in electrochemically stimulated release media considering positive (+), negative (−), or no stimuli (0) at different time points (days 2 and 4). Scale bar of 100 μm in all images.

Finally, the cytocompatibility of leachable products resulting from insulin delivery devices (NPs + insulin/e-clickPEG) after ES was verified, and cell viability was evaluated quantitatively and by means of confocal microscopy. As shown in Figure 9e, relative cell viability is higher than 80% for all the electrochemically stimulated insulin release media, regardless of electrical stimuli or time. In fact, a relative survival rate ranging from 88 to 96% was observed when applying a positive constant potential, compared to the application of a negative electrical stimulation or no stimulation, with values ranging from 81 to 96%. Overall, regardless the potential leachable and/or degradation products from the NPs + insulin/eclickPEG devices after their usage, the results demonstrate the devices to be cytocompatible and adequate to be used in a biological context, thus posing minimal risk to cells with little influence of the potential applied. Fluorescence microscopy images confirm this statement as minimal cell death was detected, with cells exhibiting a healthy density and regular morphology (Figure 10).

## 3 Conclusions

Taking advantage of the clickable nature of thiol–yne PEG-based click-hydrogels, we have prepared a soft biointerface as an optimized insulin delivery device. Most importantly, by incorporating biocompatible PEDOT NPs as conductive moieties, electroactivity was achieved to precisely control the release of insulin over an extended period of time through electrical stimulation. The design of the device was tailored according to the results from DFT calculations. The interactions between representative molecules for 3-arm and 4-arm alkyne- and thiol-functionalized PEG precursors, as well as EDOT oligomers containing eight repeating units, were studied to reveal that, even though both examined PEG-based click-hydrogels established a favorable interaction with PEDOT, the 4-arm hydrogel displayed a more stable one. Regarding the controlled delivery of insulin from the optimized device, we emphasize the following significant outcome: insulin delivery is electrochemically managed depending on the applied voltage. Specifically, insulin release is triggered by a positive voltage (+0.6 V), while insulin leakage by passive diffusion is notably diminished by a negative voltage (−0.6 V). Such twofold action allows for a more accurate and personalized insulin administration considering real-time glucose levels. In addition to this, the overall performance of the device considering mechanical performance, swelling response, stability, and biocompatibility, matches the requirements of our application. Therefore, our device, which is based on electroresponsive nucleophilic thiol–yne click-hydrogels, acts as a unique conductive polymeric matrix designed to control hyperglycemic events.

## 4 Materials and Methods

### 4.1 Materials

3,4-Ethylenedioxythiophene (EDOT, 97%), anhydrous lithium perchlorate (LiClO_4_), ammonium persulfate (APS), dodecyl benzenesulfonic acid (DBSA), ethanol, and insulin bovine with a purity ≥25 SUP/mg and *M*_w_ = 5733.49 (27 SUP = 24 IU) were obtained from Sigma-Aldrich and used without further purification. PEG precursors were synthesized as previously reported (details in the Supporting Information).^47^ Screen-printed electrodes were purchased from Metrhom (Drop Sense C110). In those, the electrochemical cell consists on a WE and an auxiliary electrode (CE) made of carbon, and a silver reference electrode. 0.1 M PBS solution was prepared using Milli-Q water at room temperature (21 °C) (pH = 7.4, 137 mM NaCl, 8 mM Na_2_HPO_4_, 2 mM KH_2_PO_4_, and 2.7 mM KCl—all salt reagents needed were purchased from Sigma-Aldrich). For the in vitro cellular biocompatibility studies, L-929 mouse fibroblasts were purchased from ATCC (number CCL-1), while low-glucose DMEM, fetal bovine serum and antibiotic–antimycotic (anti– nti) solution were acquired from Gibco, Thermo Fisher Scientific, Grand Island NY, USA. The In Vitro Toxicology Assay Kit, MTT-based (TOX1-1KT) was purchased from Sigma-Aldrich.

### 4.2 Synthesis of Poly(3,4-ethylenedioxythiophene) Nanoparticles

PEDOT NPs were synthesized by the oxidative emulsion polymerization of EDOT in an aqueous solution of ethanol (12.5 vol %).^54^ APS was employed as oxidant, whereas DBSA was utilized as an anionic surfactant to facilitate the formation of micelles, as well as doping agent. The reaction was conducted at 40 °C, under stirring (750 rpm), and protected from light. 0.115 g of DBSA were added to 31.7 mL of Milli-Q water (preheated at 40 °C) and stirred for 1 h. Then, 144 μL of EDOT was added dropwise, followed by 4 mL of ethanol. After 1 h of agitation, the solution was finally combined with 4 mL of Milli-Q water containing 0.7296 g of APS. The reaction proceeded for 16 h until it was allowed to cool down to room temperature (21 °C). The resulting PEDOT NPs were washed by centrifugation at 11,000 rpm and 4 °C for 40 min. After washing, the supernatant was removed and 40 mL of Milli-Q water were added to the NPs prior sonication for 20 min. Finally, PEDOT NPs were dried under vacuum.

### 4.3 Synthesis of Electroactive Thiol–Yne PEG-Based Click-Hydrogels (e-clickPEG Hydrogels)

In a solution of 2 mL of 0.1 M PBS, 12.4 mg of PEDOT NPs were stabilized (6.2 mg/mL) under stirring for 3 days at 1000 rpm and then sonication for 30 min. To 0.5 mL of this suspension, 4-arm PEG precursors functionalized with thiol and alkyne moieties (80 mg of 4_S_ and 75 mg of 4_A_, respectively) were dissolved separately and vortexed for 60 s. Then, both precursor solutions were mixed together (click-hydrogel solution) and vortexed for 10 s before allowing the system to gel. The resulting hydrogel was produced with a thiol/alkyne molar ratio of 1:1 and a total PEG precursor concentration of 15 wt %. Control thiol–yne PEG-based click-hydrogels (clickPEG hydrogels) were prepared following the same procedure but using 0.5 mL of 0.1 M PBS without PEDOT NPs.

### 4.4 Fabrication of Insulin Delivery Devices (NPs + insulin/e-clickPEG)

As a first step, 1.23 mg of PEDOT NPs was added to 0.2 mL of 0.1 M PBS and stabilized as previously described. After sonication, 45 mg of insulin was added and the suspension was vortexed for a few seconds. Then, 20 μL were drop-casted onto a screen-printed electrode (WE) and left to dry at room temperature (21 °C). On top of this layer (NPs + insulin), 75 μL of the click-hydrogel solution were drop-casted and let to gel and stabilize for 15 min before any further characterization or release study.

### 4.5 Insulin Delivery—Electrochemical Release and HPLC Detection

NPs + insulin/e-clickPEG devices were immersed in 2 mL of cell culture medium 0.1 M PBS solution at room temperature (21 °C) after gelation and exposed to a constant stimulation potential for 100 s a day during the first 4 days. This chronoamperometric stimulation (CA) was conducted with an Autolab PGSTAT101 (Metrohm Autolab B.V., Utrech, The Netherlands) considering three different conditions: a positive voltage (0.6 V), a negative voltage (−0.6 V), and no voltage. Every 24 h, the 2 mL of PBS solution were collected and replaced before and after the stimulation event, respectively. One mL was employed to determine insulin concentration by HPLC, while the other was reserved for cytotoxicity assays. A previously reported HPLC method has been used for the detection of insulin in a Hitachi LaChrom HPLC system with a reversed phase Nucleosil C18 column (5 μm, 250 × 4 mm, Macherey-Nagel).^61^ The ratio of acetonitrile to 0.1% TFA aqueous solution in the mobile phase was changed linearly over the course of 5 min, from 30:70 (v/v) to 40:60 (v/v). The ratio of 40:60 (v/v) was maintained during the following 5 min. The injection volume was 20 μL, the detection wavelength was 214 nm, and a flow of 1 mL/min was used. HPLC tests were conducted at room temperature (21 °C), and the insulin concentration was determined by calculating the overall peak area.

### 4.6 Characterization of NPs + Insulin/e-clickPEG Devices

The resulting insulin delivery devices based on PEDOT NPs and electroconductive thiol–yne PEG-based click hydrogels were fully characterized in terms of chemical composition, structure and morphology, mechanical and electrochemical performance, as well as swelling response. Besides, the cytocompatibilty of the system was verified. The details on each characterization technique and procedure followed can be found in the Supporting Information.

### 4.7 Theoretical Calculations of PEDOT···PEG Precursors Interactions

The strength of the interactions between PEDOT chains and thiol–yne PEG-based click-hydrogels derived from 3-arm and 4-arm alkyne- and thiol-functionalized PEG precursors was examined using DFT calculations, which were performed using the Gaussian 09 computer package.^62^ The geometries of the different investigated model complexes were fully optimized with the B3LYP^63,64^ combined with the 6-311++G(d,p) basis set. Geometry optimizations were performed in water (ε = 78.4), which was described through a simple self consistent reaction field method. More specifically, the PCM^65,66^ was used in the framework of the B3LYP/6-311++G(d,p) level to represent bulk solvent effects. No symmetry constraints were used in the geometry optimizations.

## Supporting Information Available

Details of the synthesis and characterization of PEG-based click precursors, as well as each characterization technique and procedure: chemical characterization, structural and morphological characterization, swelling response, electrochemical characterization, and mechanical characterization. Also, methodological information on cytocompatibility studies. Additional figures (PDF)
