# Supplementary material for: Electroresponsive Thiol–Yne Click-Hydrogels for Insulin Smart Delivery: Tackling Sustained Release and Leakage Control
Source: ACS Appl Polym Mater. 2024 Jul 11;6(14):8093–104. doi: 10.1021/acsapm.4c00911 (PMC11745422; doi:10.1021/acsapm.4c00911)
Supplement: Supplementary file 1 — ap4c00911_si_001.pdf [file ap4c00911_si_001.pdf]

# Supporting Information

**Electroresponsive Thiol-Yne Click-Hydrogels for Insulin Smart Delivery: Tackling Sustained Release And Leakage Control**

Helena Muñoz-Galán,[a,b] Hamidreza Enshaei, [a,b] João C. Silva,[c,d] Teresa Esteves,[c,d] Frederico Castelo Ferreira, [c,d] Jordi Casanovas,[e] Joshua C. Worch,[f] Andrew P. Dove,[f] Carlos Alemán [a, b, g,*] and Maria M. Pérez-Madrigal [a,b,*]

a. Departament d'Enginyeria Química, Campus Diagonal Besòs (EEBE), Universitat Politècnica de Catalunya · Barcelona Tech, Av. Eduard Maristany 10-14, 08019 Barcelona (Spain)
b. Barcelona Research Center for Multiscale Science and Engineering, EEBE, Universitat Politècnica de Catalunya, C/Eduard Maristany 10-14, 08019 Barcelona (Spain)
c. iBB – Institute for Bioengineering and Biosciences, Department of Bioengineering, Instituto Superior Técnico - Universidade de Lisboa, Avenida Rovisco Pais 1, 1049-001 Lisboa, (Portugal)
d. Associate Laboratory i4HB–Institute for Health and Bioeconomy at Instituto Superior Técnico, Universidade de Lisboa, Avenida Rovisco Pais 1, 1049-001 Lisboa (Portugal)
e. Departament de Química, Física i Ciències Ambientals i del Sòl, Escola Politècnica Superior, Universitat de Lleida. C/Jaume II n° 69, E-25001 Lleida (Spain)
f. School of Chemistry, University of Birmingham, University Rd W, Birmingham, B152TT (UK)
g. Institute for Bioengineering of Catalonia (IBEC), The Barcelona Institute of Science and Technology, Baldiri Reixac 10-12, 08028 Barcelona (Spain)

E-mail: carlos.aleman@upc.edu, m.mar.perez@upc.edu

## 1. Experimental Section

### 1.1. Synthesis and characterization of PEG-based click precursors

*1.1.1. Materials*

4-arm PEG-t*etrahydroxyl (molar mass 2 kg/*mol) was purchased from JenKem Technology, U.S.A. All other reagents were purchased from Sigma-Aldrich or Fisher Scientific and used without purification.

*1.1.2. Synthesis of alkyne PEG precursor* ($4_A$)

To a suspension of 4-arm PEG2k-OH (molar mass 2 kg/mol, 10 g, 5 mmol) in benzene (75 mL) and toluene (75 mL) was added 2 drops of concentrated $H_2SO_4$. The solution was heated to 80 °C with stirring to obtain a clear homogeneous solution. To this solution, propiolic acid (2.8 g, 40 mmol) was added and the solution was heated to reflux under Dean−Stark conditions. After no more water was collected in the condenser (about 20 h), the solution was allowed to cool to room temperature and the solvents were removed in vacuum. The resultant oil was dissolved in $CH_2Cl_2$ (100 mL) and washed with saturated $NaHCO_3$ solution (20 mL) and brine (20 mL). The organic phase was dried ($MgSO_4$) and stirred with charcoal (about 0.1 g) for 30 min at 40 °C. The solution was filtered through Celite 545 and the solvent was evaporated to collect the product as a clear to light yellow oil (yield 6.1 g, 61%).

*1.1.3. Synthesis of thiol PEG precursor* ($4_S$)

In a typical esterification (as described above), 4-arm PEG2k− OH (molar mass = 2 kg/mol, 10 g, 5 mmol) was esterified using 3-mercaptopropionic acid (4.2 g, 40 mmol) (yield 9.1 g, 77%).

### 1.2. Characterization techniques

*1.2.1. Chemical characterization*

FTIR spectra were recorded with a Jasco 4100 spectrophotometer. Samples were placed on an attenuated total reflection accessory (ATR) with a diamond crystal. For each sample, 64 images with a resolution of 4 $cm^{-1}$ were taken between 4000 and 600 $cm^{-1}$.

*1.2.2. Structural and morphological characterization*

To characterize morphological features, a scanning electron microscopy (SEM) was used and operated at 5 kV (Zeiss Neon40 outfitted with a focused ion beam with an energy-dispersive X-ray (EDX) spectroscopy system). To prevent electron charging issues, all samples were sputter-coated with a thin carbon layer using a K950X Turbo Evaporator. When necessary, and prior to SEM observation, samples were liophilized (Liotop 101L, Liobras). Pore size was determined from SEM micrographs and using the software SmartTIFF (v1.0.1.2.).

### *1.2.3. Physical characterization – swelling response*

Hydrogel characterization was completed by determining gel fraction (GF), equilibrium water content (EWC), and swelling factor (SF) parameters. Hydrogels were produced as described and left to cure for 1 h at room temperature (21 °C) before further testing. No washing step was conducted after gelation for any of the three characterization procedures. Accordingly, hydrogels are referred to as prepared. For measuring the SF value, as prepared hydrogels were immersed in PBS and incubated at 37 °C in an orbital shaker incubator at 80 rpm for 10 days. PBS solution was changed regularly and, at every time point, hydrogels were taken out of the solution, dried carefully, and weighted. The SF value was calculated by applying the following expression:

$$SF(\%) = \frac{W_0}{W_t} x100 \qquad \text{(Eq. 1)}$$

were $W_0$ is the initial weight before immersion (as prepared) and $W_t$ is the weight at each specific time point.

Regarding the GF value, as prepared hydrogels were lyophilized and weighted ($W_{L1}$). Afterwards, they were immersed in Milli-Q water for three days. During these three days, several washes a day were performed to clean the medium of solute concentration. After three days the rehydration process was finished, and the hydrogels were again lyophilized and weighted ($W_{L2}$). Then, the GF was calculated according to the following expression:

$$GF(\%) = \frac{W_{L2}}{W_{L1}} x100 \qquad \text{(Eq. 2)}$$

The equilibrium water content (EWC) corresponds to the amount of water in the swollen hydrogel by weight after 24 hours of immersion. As prepared hydrogels were immersed in Milli-Q water for 24 h and weighted after drying manually the water excess ($W_S$). Then, hydrogels were lyophilized and weighted again ($W_L$). The EWC value was calculated according to the following expression:

$$EWC(\%) = \frac{W_s - W_L}{W_s} x100 \qquad \text{(Eq. 3)}$$

### *1.2.4. Electrochemical characterization*

Electrochemical characterization was performed using Autolab PGSTAT101 (Metrohm Autolab B.V., Utrech, The Netherlands) by means of cyclic voltammetry (CV). All experiments were run in a 0.1 M PBS solution (pH = 7.4) at 21 °C considering −0.2 V and 1.0 V as initial / final and reversal potentials, respectively, and a scan rate of 50 mV/s. The electroactivity (*i.e.* ability to exchange charge reversibly) was evaluated by using the NOVA v2.1 software (Metrohm Autolab B.V., Utrech, The Netherlands). All characterization assays were performed considering at least three independent samples.

### *1.2.5. Mechanical characterization*

All uniaxial compressive testing was performed on a universal testing machine (EZ-Test LX, Shimadzu) fitted with a load cell of 100 N. Hydrogel samples were prepared with cylindrical shape with a diameter of 1 cm and height of 8 mm. A preload force of 0.2 N was set, and each test was carried out at a compression velocity of 1 mm/min. All compression tests were repeated 6 times and an average of the data was taken to find the Young's modulus. Data was analyzed using TRAPEZIUM-X analysis software (version 1.5.7, Shimadzu Corporation). Young's modulus was calculated from the initial 5% of the stress/strain curve.

## 1.3. Cytocompatibility studies

### *1.3.1. Culture of L-929 mouse fibroblasts*

*In vitro* cell culture assays were performed using L-929 mouse fibroblasts (ATCC number CCL-1). Cells were thawed from nitrogen cell banks and expanded in low-glucose Dulbecco's modified Eagle's medium (DMEM, Gibco, ThermoFisher Scientific, Grand Island NY, USA) supplemented with 10% v/v fetal bovine serum (FBS, Gibco, ThermoFisher Scientific) and 1% v/v antibiotic-antimycotic (Anti-Anti, Gibco, ThermoFisher Scientific) inside an incubator at 37 °C/5% $CO_2$. Cells were passaged to new tissue-culture polystyrene (TCPS) flasks when a confluence of 80-90% was reached and the culture medium was fully renewed every 3 days.

### *1.3.2. In vitro cytotoxicity testing of e-clickPEG hydrogels*

The cytocompatibility of e-clickPEG hydrogel-based devices was assessed using L-929 mouse fibroblasts and following the ISO 10993-5 and ISO 10993-12 guidelines. Before testing, the hydrogels were sterilized through incubation in a 1% v/v Anti-Anti solution (in PBS) for 3 h at room temperature (one wash every hour).

The material was evaluated both by the indirect extract test and direct contact test. For both tests, cells were seeded in 24 TCPS-plates at 1.0 x $10^5$ cells/well and cultivated to confluence for 24 h at 37 °C and 5% $CO_2$. L-929 cells cultured in DMEM + 10%FBS + 1% Anti-Anti culture media under standard conditions were used as negative control and latex was used as a positive control for cell death.

Material extracts were prepared by incubating e-clickPEG hydrogels in culture media (ratio of 3 $cm^2$ of hydrogel surface per mL) for 24 h at 37 °C and 5% $CO_2$. To do so, discal-shaped hydrogels of radius 0.45 cm and height 0.5 were immersed in 3 mL of media. The fibroblasts were exposed to the extracted medium containing the hydrogel leachable products for 72 h at 37 °C and 5% $CO_2$. Afterward, the extracted medium was removed and the MTT assay was done using the In Vitro Toxicology Assay Kit – MTT-based (Sigma-Aldrich)

following the manufacturer's instructions. Briefly, cells cultured in extracted medium and controls were incubated with an MTT (3-(4,5-dimethylthiazol-2-yl)-2,5-diphenyl tetrazolium bromide) solution at 1 mg/mL for 2 h at 37 °C. The MTT solution was then carefully removed and the resulting violet formazan crystals (resulting from the metabolic reduction of MTT by viable cells) were dissolved with a 0.1 N hydrochloride acid solution (in anhydrous isopropanol) under agitation for 5-10 min. The absorbance values of the resultant solutions were measured in a multi-plate spectrophotometer (Infinite M200 PRO, TECAN, Männedorf, Switzerland) at 570 nm. The percentage of viable fibroblasts was calculated by comparison with the values obtained for the negative control. Five sample replicates (n = 5) were tested per experimental condition, and the absorbance values of each one were read in triplicate.

In the direct contact assay, e-clickPEG hydrogels were carefully placed on top of the confluent cell monolayer of L-929 fibroblasts (three replicates per condition) and incubated for 72 h at 37 °C and 5% $CO_2$. Then, the viability and morphology of the fibroblasts were qualitatively assessed under an inverted optical microscope (LEICA DMI3000B, Leica Microsystems, Wetzlar, Germany) equipped with a digital camera (Nikon DXM1200F, Nikon Instruments Inc., Melville, NY USA).

### *1.3.3. Cytocompatibility of leachable products resulting from insulin delivery devices (NPs+INS/e-clickPEG) after electrochemical stimulation*

Before cell culture assays, the culture media obtained from the electrochemically-stimulated insulin release assay was sterilized by filtration using a 0.22 µm filter (Merck, Darmstadt, Germany). L-929 fibroblasts were seeded in 24 TCPS-plates at 1.0 x $10^5$ cells/well and cultivated to confluence for 24 h at 37 °C and 5% $CO_2$. Then, the culture medium was removed and the different electro-stimulated media (Positive (+) stimuli vs. Negative stimuli (-) vs. non-stimuli) at different time points (day 1, 2, 3, and 4 before and after stimulation) were added to the cultures and incubated for 24 h at 37 °C and 5% $CO_2$. Cells cultured in DMEM + 10%FBS + 1% Anti-Anti culture media under standard conditions were used as control. Cell viability was evaluated quantitatively using the MTT assay (n = 4 replicates, as described previously) and visualized using the LIVE/DEAD Viability/Cytotoxicity kit (Invitrogen, ThermoFisher Scientific). Briefly, the media was removed and the samples were incubated in PBS containing 2 µM calcein AM (stains live cells in green) and 4 µM ethidium homodimer-1 (stains dead cells red) for 1 h at room temperature (21 °C), washed once with PBS, and imaged under a fluorescence microscope (LEICA DMI3000B, Leica Microsystems).

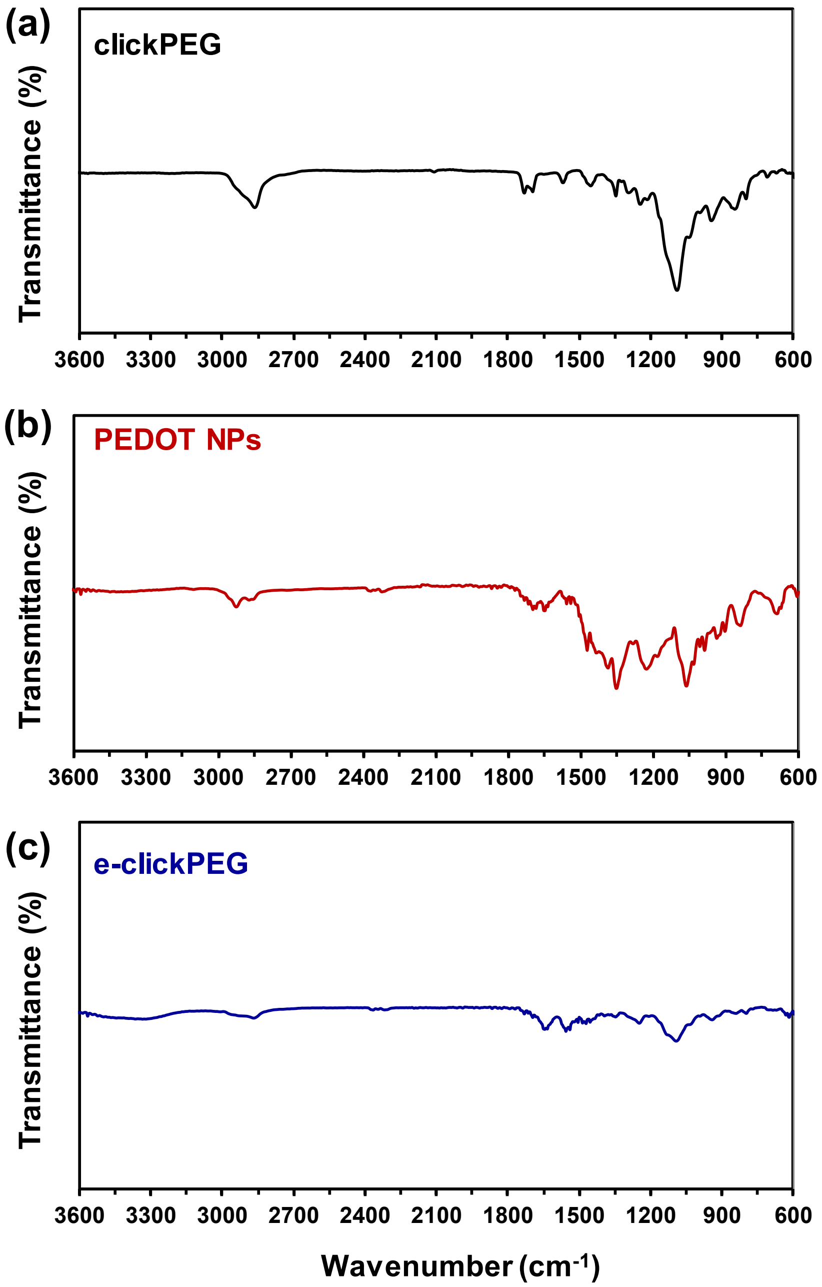


**Figure S1.** FT-IR spectra from 600 to 3600 $cm^{-1}$ obtained for (a) thiol-yne clickPEG hydrogels; (b) PEDOT NPs, and (c) e-clickPEG hydrogels.
